# Correlates of Physical Activity in Brazilian Older Adults: The National Health Survey 2019

**DOI:** 10.3390/ijerph20032463

**Published:** 2023-01-30

**Authors:** Amanda Santos da Silva, João Carlos do Nascimento Melo, Zainovan Serrão Pereira, Jullyane Caldas dos Santos, Roberto Jerônimo dos Santos Silva, Raphael Henrique de Oliveira Araújo, Ricardo Aurélio Carvalho Sampaio

**Affiliations:** 1 Postgraduate Program in Physical Education, Federal University of Sergipe, São Cristóvão 49100-000, Brazil; 2 Graduate Program in Health Sciences, Londrina State University, Londrina 86057-970, Brazil

**Keywords:** aging, epidemiology, exercise, health behavior, health surveys

## Abstract

Engagement in physical activity (PA) depends on intrapersonal, interpersonal/cultural, organizational, physical environment and political factors. Considering that it is important to understand this phenomenon in different populational contexts, this study aimed to investigate the factors related to engagement in PA according to sociodemographic aspects, eating habits, self-rated health, activities of daily living, noncommunicable diseases, mental health and public policies in Brazilian older people. This study had a cross-sectional design and used data from the Brazilian National Health Survey, 2019. Sample size was composed of 22,726 participants, aged 60 years or older, of both sexes, and all the data were collected by interview/questionnaire. According to the adjusted logistic regression, males were more active than females (OR = 1.59 (95% CI 1.40–1.80)), and those living in northern and northeastern Brazil were more likely to be inactive when compared to the southeastern region. Moreover, those with a higher educational level and income (OR = 1.36 (1.06–1.73) and OR = 1.60 (1.22–2.11)); with healthy eating habits (OR = 1.05 (1.03–1.06)); with positive self-rated health (OR = 2.67 (95% CI 1.51–4.71)); with better functional autonomy (OR = 1.22 (1.17–1.27)); and who reported that there was some public place (square, park, closed street, beach) to go for a walk, exercise or practice sport close to their home were more likely to be active (OR = 1.49 (1.34–1.67)). Sociodemographic factors, healthy eating habits, positive self-rated health, higher functioning in activities of daily living and living close to places where PA is practiced were associated with regular engagement in PA (i.e., ≥150 min/week).

## 1. Introduction

Although life expectancy has increased globally in recent decades, a healthy life expectancy remains an issue in most countries, including Brazil. It means that even though people are living longer, this process may be characterized by the presence of several diseases, such as noncommunicable chronic diseases (NCDs) including hypertension, diabetes, neoplasms, cerebrovascular and cardiovascular diseases, and functional impairments. In this sense, evidence has pointed to the regular practice of physical activity (PA) as one of the nonpharmacological strategies for its treatment and prevention, in addition to promoting a more active aging, since it allows improvements in functional levels [1].

Regular engagement in PA is complex and depends on many factors, including intrapersonal (biological, psychological), interpersonal/cultural, organizational, physical environment (built, nature) and political (laws, rules, regulations and codes) factors [2,3]. On the other hand, physical inactivity (i.e., those who fail to achieve ≥150 min of PA per week, according to the World Health Organization (WHO) [1] is associated with negative health outcomes [4]. Data from 2019 presented a prevalence of 40.3% of physical inactivity in Brazil, which worsens in older people, in which the prevalence was 59.7%, highlighting the need for actions to reverse/face this scenario [5]. As a way of suggesting assertive actions, it is important to understand the phenomenon of adherence to the practice of PA and its benefits [6,7] in the various possible populational contexts.

Most studies following this theme have been carried out in developed countries [8,9], which leads us to hypothesize that it could be inappropriate to extrapolate their findings to developing countries with great socioeconomic heterogeneity such as Brazil [10]. In addition, it is necessary to identify which factors are associated with PA in older adults, especially because this group has much to benefit from regular PA, including prevention of NCDs, diabetes, reduction in the symptoms of depression, postponement of cognitive decline and improvement of memory [11,12].

In this regard, Morais et al. (2022) [13] aimed to identify individuals’ levels of PA and associations according to intrapersonal factors, sociodemographics and public spaces for the practice of PA, verifying positive associations in relation to education. However, that study investigated a specific region, which makes it difficult to expand the conclusions to the entire Brazilian population. So far, to the best of our knowledge, studies carried out with older people have focused on examining cross-sectional associations, considering PA domains, and in specific regions of the country [14,15]. Moreover, health surveys conducted in South American countries have limited their data collection to adults, as reported by the South American Physical Activity and Sedentary Behavior Network (SAPASEN) in a multi-dataset analysis [16]. Considering this, we hypothesize that this study may provide more reliable data regarding these associations, mainly considering the representative national sample size.

Thus, robust evidence on the factors associated with PA in older adults is still scarce and inclusive in low- and middle-income countries [17]. Therefore, considering this gap, this study aimed to investigate the factors related to PA according to sociodemographic aspects, eating habits, self-rated health, activities of daily living (ADL), NCDs, mental health and public policies in Brazilian older people.

## 2. Materials and Methods

### 2.1. Study Design and Population

This is a cross-sectional study, using the National Health Survey (NHS), 2019. The NHS is a national, household-based survey carried out by the Brazilian Institute of Geography and Statistics (IBGE) in partnership with the Ministry of Health. The NHS had its first edition launched in 2013 and was updated in 2019. In addition, it aims to provide the country with information on the health determinants, conditions and needs of the Brazilian population, enabling consistent measures to be established, capable of assisting the formulation of public policies and achieving greater health intervention effectiveness [18].

Data collection organization involved interviewers (data collection agents), supervisors and coordinators from among IBGE personnel. All interviews were conducted using mobile data collection devices. A total of 108,525 private households were visited with 94,114 residents aged 15 or over being interviewed between August 2019 and March 2020, resulting in a “no” response rate of 6.4%. For the present study, information from 22,726 people, aged 60 years or older, of both sexes, were analyzed. The NHS full methodology is described by Stopa et al. (2020) [18].

This study followed the Strengthening the Reporting of Observational Studies in Epidemiology (STROBE Statement) regarding cross-sectional studies, which can be viewed at http://www.strobe-statement.org (Accessed on 20 October 2022).

### 2.2. Dependent Variable

To verify the frequency and time spent on PA, all domains were considered: occupational, domestic, leisure and transportation (i.e., occupational PA refers to carrying/lifting weights or walking a lot during the work routine; domestic PA indicates regular cleaning or heavy cleaning, gardening; leisure PA refers to the practice of a sport/physical exercise during free time; and transportation indicates commuting by walking or cycling to and from work). All the answers were related to the weekly frequency and daily duration, which were multiplied for each domain and then summed. The cutoff point was established at ≥150 min/week, considering the recommendations of the World Health Organization (WHO) [5]. Thus, individuals were considered “active” if they performed ≥150 min/week and were considered “inactive” if they performed ˂150 min/week.

### 2.3. Independent Variables

The exposure variables were presented continuously and categorically, namely: (a) sociodemographic, which consisted of information on sex, age, marital status, region of living in Brazil, educational level, race/color/ethnicity (in this study, the categorization was “White” and “non-White”, with “non-White” being people who self-declared as Black, Brown, Yellow or Indigenous—as specified by the IBGE) and per capita income; (b) eating habits; (c) perception of health status; (d) ADL; (e) NCD; (f) mental health; (g) community health agent or family health team; and (h) public places to practice PA. Detailed information on the variables is presented in Table 1.

### 2.4. Statistical Analysis

Descriptive data were presented as frequency (95% confidence interval) or mean (95% confidence interval). Binary logistic regression was used to analyze the possible associations among PA and the other investigated factors. The variables were inserted in a hierarchical model. All analyses were performed using complex sample weights in Stata^®^ software (Statistics/Data Analysis), Version 16.0. The significance level was set at 5%.

## 3. Results

The sample of this study was composed of 22,726 older people with a mean age of 70.1 (95% CI, 70.0–70.2) years. Of these, 56.0% (95% CI, 55.0–57.1) were physically inactive, while 44% (95% CI, 42.9–45.0) were active.

Regarding sociodemographic variables, about 56.7% were female, 51.3% self-reported being White, 63.7% pointed out that they had no education or incomplete elementary school, 42.2% reported an income of more than 1 salary up to 3 basic salaries, 46.4% lived in the southeast region and 51.1% declared to be married. For health-related variables, 16% reported healthy eating habits, about 41.4% reported perceiving a regular state of health and 60.1% reported having NCD. The sample characteristics are described in Table 2.

Table 3 presents the results of the binary logistic regression using the crude and adjusted analysis models. In summary, the adjusted analysis showed that males were more active than their female counterparts (OR = 1.59 (95% CI 1.40–1.80)); age was negatively associated with the chance of being active (OR = 0.94 (95% CI 0.94–0.95)); residents of the north and northeast regions were about 17% (95% CI 0.67–0.98) and 19% (95% CI 0.71–0.95) more likely to be inactive when compared to the southeastern region; those who completed higher education and had an income of more than 3 minimum wages had 1.36 (95% CI 1.06–1.73) and 1.60 (95% CI 1.22–2.11) greater chance of being active. In addition, people who had healthy eating habits; “good” and “very good” self-rated health; better functional autonomy; and who reported that there was some public place (square, park, closed street, beach) to go for a walk, exercise or practice sport close to their home were more likely to be active (OR = 1.05 (95% CI 1.03–1.06); OR = 1.69 (95% CI 1.00–2.68); OR = 2.67 (95% CI 1.51–4.71); OR = 1.22 (95% CI 1.17–1.27); and OR = 1.49 (95% CI 1.34–1.67), respectively). Reporting receiving a monthly visit from a community health agent or a member of the family health team, having NCDs and depressive disorders according to the PHQ9 did not show significant associations with the outcome.

## 4. Discussion

The aim of this study was to investigate the factors associated with being involved in regular PA and outcomes regarding sociodemographic aspects, eating habits, self-rated health, ADL, NCDs, mental health and public policies in Brazilian older adults. According to the results, the prevalence of physical inactivity was 56.0% in this population [5].

It is important to highlight the differences between the methodological characteristics used in our study, which can be identified as strengths, in comparison with other studies already conducted: (i) some variables were scored to allow a comprehensive approach in analysis; (ii) the use of the NHS data from 2019, which is the most recent version of the nationwide survey; and (iii) a wide category of variables was used to identify the factors that may influence PA.

Indeed, in the scientific literature, the factors examined as correlates or determinants of PA have expanded beyond biological factors [2,3], and sociodemographic, lifestyle, mental health and others are also considered important for the analysis in different populations and contexts.

Given this, it was found herein that sociodemographic aspects, eating habits, self-rated health, ADL and living near places to practice PA were associated with engagement in PA. On the other hand, having NCD, depression and not receiving visits from a community health agent or a member of the family health team were not associated with the outcome.

Regarding sociodemographic conditions, the variables gender, region, educational level and per capita income showed significant associations with the outcome. As expected and already reported in the literature, being male, having high levels of education and having a high per capita income are associated with being physically active. Additionally, older people were more inactive compared to younger people [19,20].

It was also observed that living in northern and northeastern regions showed the greatest differences in PA. About 17% and 19% were physically inactive when compared to those living in southeastern Brazil. These results can be emphasized from the perspective of the great socioeconomic, infrastructure, urbanization level and life expectancy disparities, especially because the southeastern area of Brazil is the country’s richest and most developed region. Such differences can reflect in the opportunity to practice PA, mainly due to aspects related to the spaces for practice, which in Brazil are mostly private facilities [21,22].

We observed that older adults with better eating habits scores were more likely to be physically active when compared to older adults with worse eating habits scores. The scores for eating habits were determined according to what participants usually eat; in this sense, food choices are influenced both by individual determinants [23], through individuals’ knowledge about food and nutrition, their perceptions of healthy habits, age and health status and by social determinants, such as income and education, social and cultural factors, which can determine the quality of their food [24]. Previous studies reinforced our findings by showing that healthy habits are closely associated with PA in several countries around the world, including Brazil [24,25].

Although dietary patterns vary between cultures, the so-called “healthy foods” are often characterized by foods that constitute the food base of the Brazilian population [26,27]. A study carried out by Romeiro et al. (2020) [26] identified that a high socioeconomic level and education were associated with greater adherence to healthy eating habits, reaffirming the importance of educational actions related to food in all cultural and social segments.

Our results showed that the better the perception of the health status of the older adult, the greater the chance of being physically active, corroborating the findings of Cardoso et al. (2008) [28]. Self-rated health is considered a very important predictor of health status in older people, as it encompasses physical, emotional, cognitive, social aspects, aspects related to well-being and life satisfaction. Therefore, through this subjective assessment, we can look at it from two perspectives; the older adults can clearly answer that they feel good about their health and, therefore, are willing to practice PA, but they can also report that they are dissatisfied and, therefore, do not want to practice any activity [29,30]. In addition, the perception of health status has been widely used in population studies because it is consistently associated with morbidity and mortality and functional decline, presenting a better way of assessing certain health conditions [5].

We observed that those people who had better ADL scores tend to be physically active when compared to their counterparts. In the scientific literature, we can observe that this result is already established [15]. Clearly, our aim is not establishing a causal association, especially because it is possible that people with dependency in basic ADL cannot practice regular PA, and even though they can do, we may hypothesize that is below recommendations. In this sense, regular practice of PA is an important way to achieve functional autonomy and should be encouraged throughout life. The development of aerobic exercise capacity, flexibility, balance, endurance and muscle strength should be prioritized according to the characteristics of this population to provide a series of specific benefits for the biopsychosocial health of older adults [31]. 

Regarding the places to practice PA, it was observed that those who know public leisure spaces such as parks and squares tend to engage in PA regularly. Previous studies showed similar results, even in different population [32,33]. Interestingly, this association may be intertwined with other factors, such as economic and social factors. Public environments for PA, such as the quality of the streets, squares and parks and even security itself, are present at the highest socioeconomic levels and areas, but not in less favored economical areas, which are the ones most in need of support [34].

Having NCD was not associated with higher levels of PA. Having NCD is a risk factor for physical inactivity in older adults, with 22.6% of those observed in this study being inactive. We may consider that some diseases may prevent the person from being physically active, such as arthritis and rheumatism [35,36], but not hypertension and/or diabetes, for example. However, even when we remove this group of diseases, the analysis performed herein remains similar. Although there is clear evidence in the literature regarding the importance of the regular practice of PA for people of all ages, especially older people with NCDs [5,14,37,38], there is a need to strengthen its continuous monitoring. In this context, regular practice and high levels of PA tend to reduce morbidity and mortality in this population.

In this study, those who reported having depression (PHQ9) did not show a significant association with PA. The findings of the present study were confirmed in a study that analyzed the PHQ and data from the NHS, 2013 [39]. However, there are contrasting results according to a systematic review and meta-analysis carried out by Pearce et al. (2022) [14], if adults with low levels of PA met the PA recommendations, about 11.5% of cases of depression could have been avoided. In addition, those who did not practice any type of PA were twice as likely to have symptoms of depression and anxiety than those who practice PA regularly [40].

The study limitations are: (i) the cross-sectional design, as it restricts the assessment of the causality of the variables and (ii) the use of PA information through a questionnaire or proxy, as it provides a greater margin of error than objectively measuring PA. Regarding the resident selected, only in the case of their not being able to answer due to physical or mental health reasons was another resident asked to answer instead. Studies based on other health surveys have demonstrated good agreement and reproducibility between information collected by means of a proxy and information provided by the people represented by the proxy [41,42]. One of the advantages of using a questionnaire is its low cost which allows the collection of data from a representative sample of the Brazilian population.

## 5. Conclusions

The results of this study showed that the related factors to weekly engagement in PA (i.e., ≥150 min/week) in Brazilian older adults were sociodemographic factors, good eating habits, having a positive perception of health, little difficulty with ADL and living close to places where PA is practiced. Longitudinal studies are recommended to demonstrate causality in this population regarding the studied factors.

## Figures and Tables

**Table 1:** Description of the variables used in this study.

Variables	Categorization Used in Analysis (When Applicable) and Reference Codes According to the National Health Survey Questionnaire
Physical activity	“0”—inactive; “1”—active. All physical activity domains were considered: occupational, domestic, leisure and transportation. Occupational physical activity refers to carrying/lifting weights or walking a lot during the work routine (questions P038, P039, P03904, P03905, P03906); Domestic physical activity indicates regular cleaning or heavy cleaning, gardening (questions P042, P04301, P04302); Leisure physical activity refers to the practice of a sport/physical exercise during free time (questions P034, P035, P03701, P03702); Transportation indicates commuting by walking or cycling to and from work (questions P040, P04001, P04101, P04102). These were multiplied according to the frequency reported (days × duration), and then summed to represent a single variable “PA”. The cutoff point of ≥150 min/week.
Age	Question C8: Age (in years).
Sex	“0”—female; “1”—male. Question C6: Sex.
Race/color/ethnicity	“0”—White people; “1”—non-White people. Question C9: Color or race. White and non-White (i.e., Black, Yellow, Brown and Indigenous peoples).
Marital status	“1”—married; “2”—divorced; “3”—widower; “4”—single. Question C11: What is your marital status?
Educational level	“1”—uneducated or incomplete junior high school; “2”—complete junior high school or incomplete high school; “3”—completed high school or incomplete university; “4”—university. Question VDD004A: Highest Education Level Reached.
Per capita income	“1”—up to half a basic salary; “2”—more than half a basic salary up to 1 salary; “3”—more than 1 basic salary up to 3 salaries; “4”—more than 3 basic salaries. Question VDF004: Per capita household income range.
Living region in Brazil	“1”—southeast; “2”—north; “3”—northeast; “4”—south; “5”—midwest. Question V0001: Federative Unit. The states were categorized according to the Brazil macro-regions.
Eating habits	Score ranging from 0 to 28. Questions P006; P00901; P018; P015: How many days a week do you usually eat beans?; How many days a week do you usually eat at least one type of vegetable (not counting potatoes, cassava or yams) such as lettuce, tomato, cabbage, carrots, chayote, eggplant, zucchini?; How many days a week do you usually eat fruit?; How many days a week do you usually eat fish? Answers ranged from 0 to 7 days a week for each question; the answers were summed and the total score was used, which ranged from 0 to 28.
Self-rated Health	“1”—poor; “2”—very poor; “3”—regular; “4”—good; “5”—very good. Question N001: In general, how do you rate your health?
Activities of Daily Living (ADL)	Score ranging from 0 to 15. Questions K004, K007, K010, K013, K016: In general, how difficult is it for you to shower alone including getting in and out of the shower or bath?; In general, what degree of difficulty do you have going to the bathroom alone including getting up and getting off the toilet?; In general, how much difficulty do you have in dressing yourself, including putting on socks and shoes, zipping and fastening and undoing buttons?; In general, how difficult is it to walk at home alone from one room to another in the house, on the same floor, such as from bedroom to living room?; In general, how difficult is it to lie down or get up from a chair by yourself? Answers ranged from 0 to 3 (0 no difficulty to 3 unable to perform); the answers were summed and the total score was used, which ranged from 0 to 15.
Noncommunicable Chronic Diseases (NCDs)	“0”—no; “1”—yes. Questions: Q00201, Q03001, Q068, Q060, Q079, Q074, Q120: Has a doctor ever diagnosed you with arterial hypertension (high blood pressure)?; Has a doctor ever diagnosed you with diabetes?; Has a doctor ever diagnosed you with a cerebral vascular accident or stroke?; Has a doctor ever diagnosed you with a high cholesterol level?; Has a doctor ever diagnosed you with arthritis or rheumatism?; Has a doctor ever diagnosed you with asthma (or asthmatic bronchitis)?; Has a doctor ever diagnosed you with cancer?. The presence of one or more NCD was considered as “1” yes.
Patient Health Questionnaire 9 (PHQ9)	Score ranging from 0 to 36. Questions: N010 to N018: In the last two weeks, how often have you had sleep problems, such as difficulty falling asleep, waking up frequently at night or sleeping more than usual?; In the last two weeks, how often did you have problems with not feeling rested and energetic during the day, feeling tired, without energy?; In the past two weeks, how often have you had little interest or no pleasure in doing things?; In the past two weeks, how often have you had problems concentrating on your usual activities?; In the last two weeks, how often did you have problems with eating, such as lack of appetite or eating much more than usual?; In the last two weeks, how often have you been slow to move or speak, or instead, have you been very agitated or restless?; In the past two weeks, how often have you felt depressed, “down” or hopeless?; In the last two weeks, how often did you feel bad about yourself, feeling like a failure or feeling that you let your family down?; In the past two weeks, how often have you thought about hurting yourself in some way or thought it would be better to be dead? Responses were reported as “1”—none, “2”—less than half the days, “3”—more than half the days, and “4”—almost every day. The sum of responses was considered in a score ranging from 0 to 36.
Community Health Agent/Family Health Team	“1” monthly; “2” every 2 months and/or 4 months; “3” once; “4” never received their visit. Question B003: In the last twelve months, how often was your home visited by a community health agent or member of the family health team?
Places to practice physical activity	“0”—no; “1”—yes. Question P046: Near your home, is there any public place (square, park, closed street, beach) to go for a walk, exercise or play sports?

The National Health Survey data are available at https://www.ibge.gov.br/en/statistics/social/health/16840-national-survey-of-health.html (Accessed on 5 May 2022).

**Table 2:** General characteristics of the sample (NHS, 2019).

Variable	All (N = 22,726)	Physically Active (N = 9456)	Physically Inactive (N = 13,270)
	Mean (95% CI) or % (95% CI)	Mean (95% CI) or % (95% CI)	Mean (95% CI) or % (95% CI)
**Age (years)**	70.1 (70.0–70.2)	67.9 (67.7–68.1)	71.8 (71.6–72.0)
**Sex**			
Female	56.7 (56.1–57.2)	49.8 (48.2–51.3)	60.0 (58.7–61.4)
Male	43.3 (42.8–43.9)	50.2 (48.7–51.8)	40.0 (38.6–41.3)
**Race/color/ethnicity**			
White people	51.3 (50.3–52.3)	52.5 (50.9–54.2)	50.3 (48.9–51.6)
Non-White people	48.7 (47.7–49.7)	47.5 (45.8–49.1)	49.7 (48.4–51.1)
**Educational level**			
Uneducated or incomplete junior high school	63.7 (62.7–64.7)	54.6 (52.9–56.3)	70.7 (69.4–71.9)
Completed junior high or incomplete high school	9.7 (9.2–10.2)	10.3 (9.4–11.3)	8.7 (8.0–9.4)
Completed high school or incomplete university	15.5 (14.9–16.1)	18.6 (17.3–19.9)	12.9 (12.0–13.8)
University	11.1 (10.4–11.9)	16.5 (15.0–17.9)	7.8 (7.0–8.5)
**Per capita income**			
Up to half a basic salary	9.9 (9.2–10.4)	7.2 (6.4–7.9)	10.2 (9.4–11.1)
More than half a basic salary up to 1 salary	31.9 (41.2–43.1)	24.7 (23.4–26.0)	36.8 (35.5–38.0)
More than 1 basic salary up to 3 salaries	42.2 (41.2–43.1)	45.8 (44.2–47.4)	39.5 (38.2–40.9)
More than 3 basic salaries	16.1 (15.1–17.0)	22.3 (20.8–23.9)	13.5 (12.5–14.5)
**Living region in Brazil**			
Southeast	46.4 (45.5–47.4)	51.9 (50.6–53.2)	43.7 (42.6–44.8)
North	6.1 (5.8–6.4)	4.6 (4.3–4.9)	6.3 (6.0–6.7)
Northeast	25.4 (24.7–26.1)	21.1 (20.2–21.5)	27.5 (26.6–28.3)
South	15.7 (15.2–16.3)	16.0 (15.2–16.8)	16.4 (15.6–17.1)
Midwest	6.4 (6.0–6.7)	6.4 (6.0–6.9)	6.1 (5.7–6.5)
**Marital status**			
Married	51.1 (50.2–52.0)	46.4 (44.9–48.0)	40.9 (39.6–42.2)
Divorced	9.0 (8.5–9.40	14.0 (12.8–15.2)	9.5 (8.8–10.3)
Widower	23.9 (23.2–24.5)	22.0 (21.3–23.9)	33.0 (31.7–34.2)
Single	16.0 (15.4–16.6)	17.0 (15.8–18.2)	16.0 (15.6–17.6)
**Eating habits (score)**	16.0 (16–16.1)	16.7 (16.6–16.8)	15.5 (15.4–15.7)
**Self-rated Health**			
Poor	2.5 (2.2–2.90	1.3 (0.8–1.8)	3.5 (3.0–3.9)
Very poor	8.2 (7.7–8.7)	4.3 (3.47–4.9)	11.3 (10.5–12.0)
Regular	41.4 (40.3–40.7)	36.4 (34.8–38.0)	45.3 (44.0–46.6)
Good	39.6 (38.6–40.7)	45.7 (44.1–47.4)	34.9 (33.6–36.1)
Very good	8.2 (7.7–8.8)	12.3 (11.2–13.3)	5.1 (4.5–5.7)
**ADL (score)**	14.0 (14.0–14.1)	14.7 (14.7–14.8)	13.8 (13.7–13.8)
**NCD**			
No	39.9 (39.3–4.6)	70.8 (69.3–72.2)	77.4 (76.3–78.5)
Yes	60.1 (59.4–60.7)	29.2 (27.8–30.7)	22.6 (21.5–23.7)
**PHQ9 (score)**	1.8 (1.8–1.9)	2.7 (2.6–2.9)	3.9 (3.8–4.1)
**Community Health Agent/Family Health Team**			
Monthly	40.8 (39.5–42.1)	39.1(37.1–41.1)	41.7 (40.0–43.4)
Every 2 months and/or 4 months	16.6 (15.7–17.6)	16.6 (15.1–18.1)	16.3 (15.0–17.5)
Once	10.9 (10.2–11.7)	10.8 (9.5–12.2)	11.2 (10.2–12.2)
Never received their visit	31.6 (30.4–32.9)	33.5 (31.6–35.4)	30.8 (29.2–32.3)
**Places to practice physical activity**			
No	48.2 (46.9–49.5)	40.3 (38.5–42.1)	54.4 (52.9–56.0)
Yes	51.8 (50.5–35.1)	59.7 (57.9–61.5)	45.6 (44.0–47.1)

Values are mean (95% CI) for age, eating habits, ADL, PHQ9 or frequency (95% CI) for all the other variables. ADL: Activities of daily living. NCD: Noncommunicable chronic diseases. 95% CI: 95% confidence interval. PHQ9: Patient Health Questionnaire 9.

**Table 3:** Associated factors with total physical activity on Brazilian older people.

Variables	Crude Model		Adjusted Model	
	Odds Ratio	(95% CI)	Odds Ratio	(95% CI)
**Sex**				
Female	Ref	Ref	Ref	Ref
Male	1.52	(1.39–1.65)	1.59	(1.40–1.80)
**Age**	0.93	(0.93–0.94)	0.94	(0.94–0.95)
**Marital status**				
Married	Ref	Ref	Ref	Ref
Divorced	1.29	(1.11–1.49)	1.14	(0.93–1.40)
Widower	0.6	(0.54–0.67)	0.95	(0.82–1.10)
Single	0.9	(0.79–1.01)	1.05	(0.89–1.23)
**Living Region in Brazil**				
Southeast	Ref	Ref	Ref	Ref
North	0.61	(0.53–0.69)	0.81	(0.67–0.98)
Northeast	0.65	(0.58–0.72)	0.83	(0.71–0.95)
South	0.83	(0.73–0.93)	0.90	(0.77–1.05)
Midwest	0.88	(0.77–1.01)	0.95	(0.89–1.23)
**Educational level**				
Uneducated or incomplete junior high school	Ref	Ref	Ref	Ref
Completed junior high or incomplete high school	1.54	(1.33–1.77)	1.15	(0.96–1.37)
Completed high school or incomplete university	1.87	(1.66–2.11)	1.06	(0.89–1.26)
Uneducated or incomplete junior high school	2.74	(2.38–3.16)	1.36	(1.06–1.73)
**Race/color/ethnicity**				
White people	Ref	Ref	Ref	Ref
Non-White people	0.91	(0.84–0.99)	1.06	(0.94–1.20)
**Per capita income**				
Up to half a basic salary	Ref	Ref	Ref	Ref
More than half a basic salary up to 1 salary	0.96	(0.83–1.11)	1.14	(0.93–1.39)
More than 1 basic salary up to 3 salaries	1.66	(1.43–1.92)	1.45	(1.18–1.78)
More than 3 basic salaries	2.37	(2.01–2.79)	1.6	(1.22–2.11)
**Eating habits**	1.05	(1.04–1.06)	1.05	(1.03–1.06)
**Self-rated health**				
Poor	Ref	Ref	Ref	Ref
Very poor	1.01	(0.67–1.53)	0.9	(0.52–1.55)
Regular	2.13	(1.46–3.11)	1.41	(0.85–2.35)
Good	3.48	(2.38–5.09)	1.69	(1.00–2.86)
Very good	6.36	(4.28–9.47)	2.67	(1.51–4.71)
**ADL**	1.31	(1.26–1.37)	1.22	(1.17–1.27)
**NCD**				
No	Ref	Ref	Ref	Ref
Yes	1.42	(1.29–1.56)	1.11	(0.98–1.27)
**PHQ9**	0.94	(0.93–0.95)	0.99	(0.98–1.01)
**Community Health Agent/Family Health Team**				
Monthly	Ref	Ref	Ref	Ref
Every 2 months and/or 4 months	1.09	(0.94–1.26)	1.02	(0.87–1.19)
Once	1.03	(0.86–1.24)	0.95	(0.77–1.16)
Never received their visit	1.16	(1.03–1.31)	0.95	(0.83–1.09)
**Places to practice physical activity**				
No	Ref	Ref	Ref	Ref
Yes	1.77	(1.62–1.93)	1.49	(1.34–1.67)

95% CI: 95% Confidence interval. Ref: Reference for analysis. ADL: Activities of daily living. NCD: Noncommunicable chronic diseases. PHQ9: Patient Health Questionnaire 9.

## Data Availability

The National Health Survey data are available at https://www.ibge.gov.br/en/statistics/social/health/16840-national-survey-of-health.html (Accessed on 5 May 2022).
